# Seeking and Exploring Efficient Ways to Target Cancer

**DOI:** 10.3390/cells9092117

**Published:** 2020-09-17

**Authors:** Tuula Kallunki

**Affiliations:** 1 The Invasion and Signaling Group, Cell Death and Metabolism, Center for Autophagy, Recycling and Disease, Danish Cancer Society Research Center, Strandboulevarden 49, 2100 Copenhagen, Denmark; tk@cancer.dk; Tel.: +45-35-257-746; 2 Department of Drug Design and Pharmacology, Faculty of Health and Medical Sciences, University of Copenhagen, 2200 Copenhagen, Denmark

Anti-cancer treatments have never been so numerous and so efficient. As a result, the number of cancer survivors has been steadily increasing. At the same time, we are facing the challenge of a decade with increasing cancer incidence, which is expected to increase by 80% in middle and low-income countries and 40% in high-income countries from 2008 to 2030 [1]. In 2008, 7.6 million people died of cancer, and with the current trend, it is estimated that by 2030 the annual cancer death rate will reach 13 million. It is unlikely that this raise will stop there. Now is the time to focus on efforts to stop this development; obviously not only more research, but also more focused cancer research is needed to discover and develop even better anti-cancer treatments. All the “easy” drugs have been discovered and thus all innovative, “out of the mainstream” scientific ideas to target cancer should be thoroughly studied. For this, we need co-operation between cancer researchers, politicians, patients and their families, and at the same we need to retain open and curious minds to explore new ideas and to find new and better ways to revisit the old ones. To obtain optimal individual benefit, cancer treatments would need to be personalized with the most suitable drugs, drug combinations and doses, which requires more research, resulting in an increased understanding of cancer biology. This will allow us to find new therapeutic target molecules and refine our understanding of the old ones. In this issue, we show insights into some promising as well as some possible drug/treatment targets, as well as the means to target them, and address some novel and unconventional ideas and approaches.

Immunotherapy, and especially different ways to boost patient immune systems to detect and fight cancer, has become an efficient way to treat cancer, showing great promise and potential against often otherwise non-curable cancers. In the review by Lichtenstern et al. [2], the authors discuss the development of the immunotherapy options for colorectal cancer (CRC), which is one of the most common and most lethal cancer types and is especially problematic to treat when detected at advanced stages. Despite scientific and clinical advances in terms of new treatment options, the five-year survival rate for metastatic CRC is still only about 14%. Immunotherapy has risen as a promising treatment against metastatic CRC due to the typically high mutational burden of this cancer type. An especially promising approach is immune checkpoint inhibition (ICI) when administered to mismatch repair deficient and microsatellite instability high (dMMR/MSI-H) CRC tumors. However not all dMMR/MSI-H tumors respond to ICI, and as of yet, no response is seen in mismatch repair proficient and microsatellite instability low (pMMR/MSI-L) tumors. An additional challenge is the gut microbiome, which has been implicated as a cause of variation in response rates to ICI depending on the microbe strain and cancer types. The poor treatment response to date has led to the necessity for the identification of new targets that could be used in combinatorial treatments such as KRAS(G12C) via its specific inhibitor AMG 510.

It is known that solid tumors can often be difficult to treat with targeted therapeutic intervention strategies such as antibody–drug conjugates and immunotherapy. This is especially true for tumors with a low mutation burden, which are often not antigenic enough to be targeted by immunotherapy. In their review article, Khazamipour et al. [3] point out how good anti-cancer treatment targets in solid tumors need to be differentially over-expressed in primary tumors and metastases in contrast to healthy organs. With this respect, they set their focus to oncofetal chondroitin sulfate, which is an excellent anti-cancer treatment target due to its virtue of being a cancer-specific secondary glycosaminoglycan modification to proteoglycans that are often expressed in various solid tumors and metastases. This is discussed as an opportunity to curb childhood solid tumors.

Cancer cell metabolism differs from that of normal cells. Tumors commonly activate metabolic pathways that upregulate nutrient synthesis and intake. Magaway et al. [4] discuss in their review the targetability of one of the central signaling metabolic pathways upregulated in tumors, the mammalian target of rapamycin (mTOR) pathway. By sensing the intracellular nutrient status, mTOR controls metabolic reprogramming via nutrient uptake and flux through various additional metabolic pathways. This makes it a promising target for anti-cancer therapy. Numerous clinical trials are ongoing to evaluate the efficacy of mTOR inhibition for cancer treatment and analogs of rapamycin, a natural mTOR inhibitor, have been approved to treat specific types of cancer. Since rapamycin does not fully inhibit mTOR kinase activity, new compounds have been engineered to better inhibit the catalytic activity of mTOR in order to more potently block its functions. Early clinical trial results of these second generation mTOR inhibitors point towards increased toxicity combined with modest antitumor activity. Magaway et al. further discuss how one of the problems encountered in these studies is the plasticity of metabolic processes. Thus, identifying metabolic vulnerabilities in different types of tumors could present opportunities for rational therapeutic strategies. A novel application of mTOR inhibitors is in the possible improvement of immunotherapeutic strategies. Using mTOR inhibitors to improve cancer vaccination strategies can also have a major impact towards their further development.

Extracellular signal-regulated kinases (Erks) encompass another kinase family that is often activated in cancers. Erks possess unique features that make them differ from other eukaryotic protein kinases. Unlike others, Erks do not autoactivate and they manifest no basal activity. They are activated as unique targets of the receptor tyrosine kinases (RTKs)–Ras–Raf–MEK signaling cascade, which controls numerous physiological processes and is mutated in most cancers. Smorodinsky-Atias et al. [5] discuss in their review how Erks have been long considered to be immune to activating mutations. Nevertheless, several such mutations have been generated in laboratory conditions and the number of mutations identified in Erks has dramatically increased following the development of Erk-specific pharmacological inhibitors and the identification of mutations that cause resistance to these compounds. Several Erk mutations have also been recently found in cancer patients. In their review Smorodinsky-Atias et al. summarize the impressive number of mutations identified in Erks so far, describe their properties, and discuss their possible mechanism of action. They discuss the possibilities to develop isoform-specific inhibitors that would specifically target Erk1 and its mutated versions. In future precision medicine, Erk1 mutations that cause drug resistance could already be taken into consideration when a therapeutic strategy is planned, and then specific drugs could be applied according to the mutation that appears in the tumor.

Multidrug resistance is a serious problem in cancer and targeting multidrug resistance by re-sensitizing resistant cancer cells is one of the big challenges in cancer biology. Among the key multidrug resistance mediating proteins are the ATP-binding cassette (ABC) transporters and the breast cancer resistance protein (BCRP). ABC transporters are plasma membrane-bound proteins that transport nutrients into cells and unwanted toxic metabolites out of cells. Cancer cells can utilize this function for transporting cancer drugs out of the cells. Several attempts to target ABC transporters to gain control in cancer have been reported, but thus far none of the inhibitors developed have been clinically approved. Ambjorner et al. [6] describe their studies on a novel BCRP inhibitor, SCO-201, which directly binds BCRP and thus prevents its function. In this study, the authors provide evidence of the specific, potent and non-toxic effects of SCO-201 in reversing the BCRP-mediated resistance in cancer cells and bringing hope to its use in the clinic.

Another membrane bound protein family with important function in cancer is the vacuolar H^+^ATPase (V-ATPase) that is located at lysosomal membranes, but can also be found at the plasma membranes of cancer cells that exhibit increased metabolic acid production, which makes them dependent on increased net acid extrusion. An acidic microenvironment favors cancer cell proliferation and survival and promotes their invasion. V-ATPase consists of at least 13 subunits, of which Flinck et al. [7] has identified ATP6V0a3 (one of the six V0 transmembrane subunits) as a negative regulator of migration and invasion of pancreatic ductal adenocarcinoma cells, where a3 is mainly found in lysosomal membranes and consistent with its mainly lysosomal role, a3 knockdown does not decrease net acid extrusion. Interestingly, Flinck et al. demonstrated that ATP6V0a3, but not the whole V-ATPase, was upregulated in pancreatic adenocarcinoma cells in comparison to pancreatic ductal epithelial cells, suggesting an additional role for it in cancer and presenting a possibility that its upregulation could be used to inhibit the invasion of these cancer cells.

In their review, Peulen et al. [8] discuss ferlins, which are phospholipid-interacting proteins involved in membrane processes such as fusion, recycling, endocytosis and exocytosis. The expression of several ferlin genes is described as altered in several tumor tissues; specifically, myoferlin, otoferlin and Fer1L4 expression have been negatively correlated with patient survival in some cancer types. Targeting myoferlin using pharmacological compounds, gene transfer technology, or interfering RNA could be considered as a novel therapeutic anti-cancer strategy, since several correlations link ferlins (and most particularly myoferlin) to cancer prognosis. However, further investigations are still needed to discover the direct link between myoferlin and cancer biology. Encouragingly, many indications suggest that myoferlin depletion can interfere with growth factor exocytosis, surface receptor fate determination, exosome composition, and metabolism.

The vast majority of cancer deaths are caused by the primary tumor metastasizing into other organs. Invasion is a prerequisite for metastasis formation, and for this reason, the inhibition of invasion could efficiently prevent metastasis formation. For this, targeting the molecules regulating invasion could be useful. One of these is an oncogenic transcription factor, myeloid zinc finger 1 (MZF1), as reviewed by Brix et al. [9]. P21 activating kinase 4 (PAK4) is another kinase that is often activated in cancers. It is also a kinase that can activate MZF1 by phosphorylating it in response to ErbB2 activation. PAK4 is considered to be a good target for the treatment of a variety of solid cancers, including breast cancer, where its inhibition for this purpose has been patented, and PAK4 inhibitors have reached clinical trials. The identification of MZF1 as an oncogenic target of PAK4, whose activity is important for the invasiveness of ErbB2 positive breast cancer cells, suggests that PAK4 inhibitors might be useful for the treatment of cancers whose aggressiveness depends on MZF1. In general, more research is still needed to increase our understanding of the detailed function of MZF1 in cancer, of the cellular cancer-promoting programs it regulates, the cancers where its inhibition would be most beneficial, and how it should be achieved.

Nuclear protein localization protein 4 (NPL4) functions as an essential chaperone that regulates microtubule structures when a cell re-enters interphase. Majera et al. [10] provide evidence of targeting of NPL4 by disulfiram (tetraethylthiuram disulfide, DSF), a drug commercially known as Antabus and commonly used for alcohol-aversion. DFS metabolizes rapidly to a diethyldithiocarbamate–copper complex, CuET, which is highly toxic to cancer cells via a mechanism that is likely to involve the immobilization and inactivation of NPL4 via its CuET-induced aggregation. CuET is especially toxic to cells that are lacking functional breast cancer genes 1 and 2 (BRCA1 and BRCA2), interfering with their DNA replication and causing replication stress. While the exact mechanism of how DSF kills BRCA1 and 2 deficient cells is not known, it is likely to involve increased NPL4–CuET-induced, supra-threshold DNA replication stress and the concomitant CuET-induced activation of the ATR-Chk1 signaling pathway.

Cancer stem cells (CSCs) are often responsible for therapeutic resistance. The study by Choi et al. [11] presents sulconazole—an antifungal medicine in the imidazole class—as a way to inhibit cell proliferation, tumor growth, and CSC formation. Part of this effect could be via the transcription factor nuclear factor kappa-light-chain-enhancer of activated B cells (NF-κB), whose expression level is decreased upon sulconazole treatment, leading to the decreased expression of interleukin-8 (IL-8). Sulconazole treatment also reduces the number of cells expressing CSC markers (CD44^high^/CD24^low^) as well as aldehyde dehydrogenase (ALDH), among others, suggesting that additional factors can be targeted by sulconazole. NF-κB/IL-8 signaling is important for CSC formation and may be an important therapeutic target for treatment targeting breast cancer stem cells.

The formation of three-dimensional (3D) multicellular spheroids (MCS) in microgravity, mimicking tissue culture conditions, was used by Melnik et al. [12] as a method for growing the metastatic follicular thyroid carcinoma cell line (FTC)-133. For this study, they utilized a random positioning machine with which the cells can be induced to detach from an already established cellular network to form 3D spheroids, which were then used as an ex vivo model system to mimic micro-metastases formation. In these conditions, the authors provide evidence that dexamethasone can prevent spheroid formation, which has previously been reported to involve NF-κB, but actually most likely functions via several different unidentified target molecules, suggesting that instead of one target, several targets should be inhibited.

A study by Gruber et al. [13] on the other hand was conducted on 278 primary tumor samples from patients with resectable esophageal cancer. This study identifies ALL1 fused gene from chromosome 1q (AF1q), a cofactor for the transcription factor 7 (TCF7), as a nuclear protein that links two important oncogenic signaling pathways activated in many cancers: WNT and signal transducer and activator of transcription 3 (STAT3). Its expression is increased in cancer progression and it is connected to the increased expression of WNT and STAT3 targets cluster of differentiation 44 (CD44) and tyrosine 705 phosphorylated STAT3 (pYSTAT3) in esophageal cancer. Patients with AF1q-positive esophageal cancer relapsed and died earlier than those with AF1q-negative disease, suggesting that AF1q could act as a cofactor to boost the transcription of CD44 and pYSTAT3, and thus implicating its involvement in the regulation of the aggressiveness of esophageal cancer and identifying it as a potential novel target molecule.
